# Novel xeno-free human heart matrix-derived three-dimensional scaffolds

**DOI:** 10.1186/s12967-015-0559-0

**Published:** 2015-06-18

**Authors:** Dolly Holt-Casper, Jeff M Theisen, Alonso P Moreno, Mark Warren, Francisco Silva, David W Grainger, David A Bull, Amit N Patel

**Affiliations:** Division of Cardiothoracic Surgery, Department of Surgery, University of Utah, Salt Lake City, UT 84112 USA; Nora Eccles Harrison Cardiovascular Research and Training Institute, University of Utah, Salt Lake City, UT 84112-5000 USA; Department of Bioengineering, University of Utah, Salt Lake City, UT 84112 USA; Department of Pharmaceutics and Pharmaceutical Chemistry, University of Utah, Salt Lake City, UT 84112 USA; University of Utah, 30 N 1900 E SOM 3c127, Salt Lake City, UT 84132 USA

**Keywords:** Scaffolds, Regeneration, Cellular transplantation, Cardiac ischemia, Cardiomyocytes, Viability, Tissue engineering, Cell signaling

## Abstract

**Rationale:**

Myocardial infarction (MI) results in damaged heart tissue which can progress to severely reduce cardiac function, leading to death. Recent studies have injected dissociated, suspended cardiac cells into coronary arteries to restore function with limited results attributed to poor cell retention and cell death. Extracellular matrix (ECM) injected into damaged cardiac tissue sites show some promising effects. However, combined use of human cardiac ECM and cardiac cells may produce superior benefits to restore cardiac function.

**Objective:**

This study was designed to assess use of new three-dimensional human heart ECM-derived scaffolds to serve as vehicles to deliver cardiac-derived cells directly to damaged heart tissue and improve cell retention at these sites while also providing biomechanical support and attracting host cell recruitment.

**Methods and Results:**

ECM-derived porous protein scaffolds were fabricated from human heart tissues. These scaffolds were designed to carry, actively promote and preserve cardiac cell phenotype, viability and functional retention in tissue sites. ECM scaffolds were optimized and were seeded with human cardiomyocytes, cultured and subsequently implanted ex vivo onto infarcted murine epicardium. Seeded human cardiomyocytes readily adhered to human cardiac-derived ECM scaffolds and maintained representative phenotypes including expression of cardiomyocyte-specific markers, and remained electrically synchronous within the scaffold in vitro. *Ex vivo*, cardiomyocyte-seeded ECM scaffolds spontaneously adhered and incorporated into murine ventricle.

**Conclusions:**

Decellularized human cardiac tissue-derived 3D ECM scaffolds are effective delivery vehicles for human cardiac cells to directly target ischemic heart tissue and warrant further studies to assess their therapeutic potential in restoring essential cardiac functions.

**Electronic supplementary material:**

The online version of this article (doi:10.1186/s12967-015-0559-0) contains supplementary material, which is available to authorized users.

## Background

Ischemic heart disease is currently the leading cause of death in the United States. Yearly, ~525,000 Americans will experience their first myocardial infarction (MI) and 190,000 will have a recurrent attack [1]. During MI, blood flow to a section of the heart is cut off, subsequently contributing to local acute tissue ischemia and the death of approximately one billion cardiomyocytes [2]. Furthermore, the resulting necrotic tissue acute zone is typically replaced by dense fibrous scar, impeding cardiac tissue regeneration and restoration of normal heart function. Due to limited intrinsic repair potential exhibited by the heart, much research has been directed towards improved methods for cardiac tissue regeneration. Towards this end, cell therapy strategies—including stem cell delivery—are now actively developed, with some showing promising results for restoring cardiac function [3, 4]. Clinical trial data has shown a reduction in myocardial scarring, a degree of healthy myocardial regeneration, or an increase in left ventricular ejection fraction in response to post-infarction cell therapy [5–7]. However, cell survival, engraftment, and control of cell differentiation state are major challenges associated with the local injection of cell populations for heart repair [8]. Additionally, cells injected after MI encounter a harsh environment that promotes apoptosis, and the majority of injected cells die within 4 days [9]. Furthermore, cells delivered through systemic circulation or coronary arteries have low rates of localization to the infarcted myocardium [9, 10], and it has been reported that only 10–15% of transepicardially injected cells are retained in the myocardium [11]. The majority of cells are lost to filter organs such as the liver and spleen [12]. These limitations must be addressed to increase treatment efficacy.

Autologous human myoskeletal cell suspensions injected into human cardiac MI sites have produced spontaneous arrhythmias [13, 14]. This pathology plus the long-standing inability to retain sufficient amounts of injected cells in MI tissue sites has prompted increasing use of biomaterials-based approaches to such cardiac tissue cellular therapeutic strategies. Notably, cell delivery modalities include gel-based injectable cell-containing carriers [15] and living cell sheets to retain cells at the injured site. The Okano group pioneered beating contiguous cardiomyocyte cell sheets for use as heart patches [16] and demonstrated their utility in porcine [17] and rat [18] models. Bel et al. also used cell sheets, but found a multilayer construct with increased ECM content provides better efficacy [19]. Chein’s group isolated progenitors that could ultimately be used to create 3D viable cell constructs that could regenerate a single portion of a damaged heart [20]. However, these groups have focused on cellular delivery, rather than on exploiting autologous extracellular matrix (ECM) either as a bulking agent or a therapeutic cell carrier. ECM proteins have also been used to regenerate damaged myocardium post-MI. Cardiac ECM has significant influence in cardiac development, and undergoes several changes during cardiac disease progression [21, 22]. ECM proteins provide essential biochemical and mechanical cues to guide cell differentiation and phenotype [23]. Although the influence of extracellular matrix in cardiac regeneration is not well understood, many studies now begin to examine this relationship. For example, ECM isolated from decellularized skeletal and cardiac muscle has been shown to improve cardiomyocyte differentiation and maturation [24], and periostin was found to increase myocyte proliferation and enhance cardiac repair [25]. Another study showed that isolated fetal cardiac ECM promoted myocyte adhesion and expansion better than adult cardiac ECM [26]. Significantly, extracellular matrix materials have been used in a variety of cardiac tissue engineering approaches. For instance, decellularized cardiac ECM in the form of an injectable hydrogel has been shown to preserve post-MI cardiac function in preclinical animal studies [27, 28]. Furthermore, decellularized cardiac biomaterials have been used as acellular patches for repair of ventricular defects in animal models [29, 30], and several groups are investigating use of cardiac ECM to promote cardiac phenotypes in 3-dimensional (3D) constructs in vitro [31–33]. These studies indicate that cardiac-derived extracellular matrix materials have a significant impact on heart regeneration. However, these 3D scaffolds are made of decellularized cardiac tissue or hydrogels, both of which have limited geometric and mechanical properties.

In addition to biochemical stimuli from ECM materials, mechanical properties and scaffold geometry may be equally important for promoting mature cardiac tissue. For instance, materials of appropriate stiffness (~10 kPa) [34] and materials patterned to promote cardiomyocyte orientation [35] have both been reported to improve cardiomyocyte differentiation and maturation. Though cardiac ECM hydrogels have been researched, they inherently lack sufficient mechanical properties [36]. Furthermore, for 3D tissue engineering constructs, high scaffold porosity enables cell infiltration and transport of nutrients and wastes [34, 37]. However, decellularized ECM biomaterials have inherently fixed porosity (constituting the absent cells), density and geometry, dependent entirely upon the organ in which it was retrieved.

Although many decellularized extracellular matrix materials are clinically available, they are relatively limited in that their mechanical properties and porosity are dependent on the tissue of origin and these properties are not easily modified. Alloderm^®^ and Matristem^®^ are two such decellularized clinical biomaterials made from human dermis and porcine urinary bladder tissue, respectively [38]. The pores of these fibrous ECM materials arise from the removal of the original cells, and the small size of these pores, on the order of 10 microns or less [39], limits cell infiltration [38]. CorMatrix ECM™, decellularized porcine small intestinal submucosa, used for pericardial and cardiac tissue repair [40], is also limited by its small pore size. Other approaches for 3D tissue engineering suffer from similar issues. Lyophilization of ECM materials, a process used to produce porous protein foams, leads to a wide distribution of pore sizes, ~10–250 μm with an average around 70 μm [41]. Additionally, electrospun scaffolds have been shown to have small pore sizes that hinder cell infiltration [42]. Taken together, the limited porosity control of tissue engineering scaffolds, especially for decellularized biomaterials, limits recellularization into their interior and eventual homogeneous growth. Furthermore, many of the clinically available decellularized materials are not of human origin, and, importantly, none are composed of tissue-specific, cardiac-derived ECM, intuitively the most relevant compilation of proteins for cardiac-specific regeneration and repair.

Based on these limitations of tissue engineering, fabrication of allogeneic cardiac-derived extracellular matrix scaffolds with well-defined structure and large pores that allow for greater cell density should be both unique and highly advantageous for cardiac tissue engineering strategies. Such scaffolds may be used to deliver cells directly to an injury site, which has shown efficacy [19], and to provide cells with a favorable microenvironment and biochemical cues, improve cell survival and retention, and to ultimately promote improved myocardial regeneration. Importantly, efficacious scaffolds must be able to support viability in the presence of diffusion alone until host vascularization can occur. Otherwise, all implanted scaffolds will necrose prior to engraftment. Towards this aim, we have decellularized human myocardial tissue using a gentle decellularization procedure to maximally preserve endogenous protein content, created ECM scaffolds with a large, controlled, interconnected pore structure, and recellularized these scaffolds with human primary cardiac cells and human iPSC-derived cardiomyocytes. This complete, human heart-derived ECM carrier (1) utilizes the intrinsic regenerative capacity of both ECM and cardiac cells in concert, (2) uniquely retains mechanical and biochemical features of native cardiac ECM, (3) can deliver many millions of viable synchronously beating cells directly to cardiac tissue sites, and (4) can support viability for time periods sufficient to become vascularized in vivo.

## Methods

### Heart ECM purification

Human heart tissue from several donors was procured after consent under IRB numbers 35241 and 35242 from the University of Utah and frozen at −80°C until the decellularization process began. Heart tissue was pooled and decellularized using a gentle immersion decellularization process, and handled in a biosafety cabinet throughout the purification process to maintain sterility. After thawing tissue overnight at 4°C, tissue was minced into pieces ~1 cm^3^ in size. Minced tissue was rinsed with sterile water (Baxter, Deerfield, USA) to remove excess blood and fluids. Next, tissue was blended in sterile water (blender model 7010S, Waring Commercial, Torrington, USA) to create a homogenous slurry. Soluble and insoluble portions of tissue were separated by centrifugation at 3,000 rcf and 40°C (Sorvall Legend XTR centrifuge, Thermo Fisher Scientific, Pittsburgh, USA). Following centrifugation, the supernatant was discarded, new sterile water was added in a 1:4 pellet to water ratio, and the pellet was dispersed and mixed thoroughly. This slurry was centrifuged again, the water was exchanged, and water washes continued until the supernatant had minimal color. Following water washes, the tissue was then washed three times in isopropyl alcohol (Sigma Aldrich, St Louis, USA), following the same procedure. Next the tissue was washed in 3 M NaCl (Sigma Aldrich); for this and the remaining washes, centrifugation was performed at 4°C. Tissue was then washed five times with 40 mg/ml sodium deoxycholate (SDC, Sigma Aldrich); with each SDC wash, the tissue was placed on an orbital shaker (model 980001, VWR, Radnor, USA) for at least 18 h before centrifugation. If after five SDC washes the SDC supernatant had color indicative of incomplete washing, additional SDC washes were performed until there was no additional color in the supernatant. Following SDC washes, tissue was washed again with 3 M NaCl, then with 100 U/ml DNase (Worthington Biochemical, Lakewood, USA) to aid DNA removal, and next with PBS (Life Technologies, Carlsbad, USA). Finally, the tissue preparation was washed ten times in sterile water. Before each new washing solution, the tissue was washed twice in sterile water to rinse out the old solution. The entire process was ~10 days in duration with the majority of time taken by the SDC wash. The resulting insoluble material, mainly composed of extracellular matrix protein components, was frozen in sterile water and lyophilized (FreeZone 2.5, Labconco, Kansas City, USA), then stored at −20°C until use.

Prior to use in scaffold fabrication or submission for mass spectrometry, lyophilized decellularized cardiac tissue was solubilized in sterile 0.1 M acetic acid (Thermo Fisher Scientific, Pittsburgh, USA) at a concentration of 5 mg/ml. After sitting in acetic acid overnight at 4°C, the decellularized tissue was homogenized (Polytron 1200 E, Kinematica AG, Littau-Lucerne, Switzerland) for 5 min. The solution was kept on ice during homogenization to prevent denaturation at high temperature.

### Scaffold fabrication

Porous ECM scaffolds were fabricated from decellularized cardiac tissue using a sacrificial polycaprolactone (PCL) porous scaffold as a template. The PCL scaffold was generated as a template for the ECM protein and later dissolved away. To create the sacrificial porous scaffold, PCL (Sigma Aldrich) was dissolved in acetone (Sigma Aldrich) at 50°C at a concentration of 0.15 g/ml. Next, water was added dropwise to 8% of the total volume. The PCL/acetone/water solution was then mixed with the porogen NaCl, sieved to select for salt crystals between 425 and 500 µm (for stem cell and primary cardiac cell ECM scaffolds) or <250 µm (for iCell iPSC cardiomyocyte ECM scaffolds). This mixture was added to 50 ml conical tubes (BD Biosciences, Franklin Lakes, USA) thoroughly mixed and centrifuged at 500 rcf and 35°C to create a uniform saturated salt suspension. The mixture was placed at −20°C to solidify, and the salt porogen was subsequently removed by immersion in excess water. The resulting PCL foam exhibits a well-defined pore structure with larger pores formed by the salt porogen and smaller pores formed by water. These foams were cut to a thickness of 0.65–0.7 mm (stem cell and primary cardiac scaffolds) or 0.3 mm (for iPSC cardiomyocyte scaffolds) using a Centaur Deli Slicer (model 212, Lombard, USA). Sliced porous PCL scaffolds were cut with biopsy punches (Miltenyi, Bergisch Gladbach, Germany) into 3 mm diameter slices intended for further studies.

Decellularized and solubilized cardiac ECM was then coated onto these porous PCL scaffolds. PCL scaffolds were immersed in a turbid solution of ECM solubilized in 0.1 M acetic acid. The PCL foam and solution were placed in a vacuum chamber (Space Saver Vacuum Desiccator, Bel-Art, Wayne, USA) to remove gas pockets and facilitate full penetration of the solution into the PCL pores. Once all air bubbles were eliminated, scaffolds were air dried in a biosafety cabinet, forming a single, contiguous coating. This process was repeated to create a total of five ECM coats. Following coating, the bulk PCL matrix was removed by dissolution in 95% acetone at 40°C for 2 days. Acetone was replaced twice daily. Scaffolds were then transferred into 95% ethyl alcohol (Decon Labs, King of Prussia, USA) and then slowly exchanged into sterile water using several serial solutions of decreasing ethanol content (70, 50, and 25% ethyl alcohol). Finally, the PCL-free scaffolds were washed 10 times in sterile water. The resulting scaffolds, comprising porous, templated decellularized cardiac tissue, were frozen in 100% water, lyophilized, sterilized with ethylene oxide gas (University of Utah Hospital, Salt Lake City, USA), and stored at −20°C until use.

### Primary derived cardiac cells

Human hearts were collected after research consent was obtained, under approved institutional review board of the University of Utah, IRB numbers 35241 and 35242. Left ventricle tissue from multiple donor hearts was mechanically minced into 1 mm^3^ pieces and placed in Dulbecco’s phosphate buffered saline DPBS(–) for 30 min. Minced tissue was placed into DPBS(–) containing 0.45 mg/ml collagenase (Worthington Biochemical Corp.) and 1 mg/ml pancreatin (Life Technologies, Carlsbad, USA). Digestion was carried out for 60 min at 37°C with gentle shaking every 5 min. Equal volume of DPBS(–) containing 10% XcytePLUS™-Xenofree Media (iBiologics, Phoenix, USA) was added to the digestion, then filtered using a 100 µm filter. Filtered cells were then washed 3× with DPBS(–) containing 10% XcytePLUS™ by centrifugation and plated into tissue culture plates (Nunc, Thermo Fisher Scientific, Pittsburgh, USA) for differential adhesion overnight at 5% CO_2_/37°C. After 12 h, non-adherent cells were discarded and adherent cells, likely consisting of a heterogeneous mixture of cardiomyocytes and fibroblasts, were passaged using TryplE (Life Technologies, Carlsbad, USA) and frozen till use using CryoStore CS10^®^ (BioLife Solutions, Bothell, USA).

### Induced pluripotent stem cell-derived cardiomyocytes

Pure human iCell cardiomyocytes containing monomeric red fluorescent protein (RFP) expressed under control of the endogenous Myh6 promoter were purchased from Cellular Dynamics (Madison, USA) and cultured according to manufacturer’s instructions. Briefly, immediately upon receipt, the vial of iCell cardiomyocytes was washed, cells were counted using a hemocytometer (Baxter, Deerfield, USA) and then resuspended at a concentration of 10 × 10^6^ cells/100 µl, which in our hands yielded 100% confluence (data not shown). Cells were seeded onto scaffolds (vide infra) and placed into iCell cardiac plating medium for 48 h at 37°C and 7% supplemental CO_2_. After 48 h, media was replaced with iCell cardiac maintenance media that was changed every 48 h throughout the duration of the experiment.

### Scaffold seeding

Lyophilized and sterilized porous ECM scaffolds were added to a 50 ml conical tube containing cell suspensions of 10^6^ cells/100 l for primary cardiac cells (on our scaffolds and Matristem^®^, Acell, Columbia, USA), and 107 cells/100 l for iPSC cardiomyocytes (on our scaffolds), and allowed to rehydrate for 10 min. During this time, a spatula was used to immerse the buoyant scaffolds continuously in the cell slurry. Air bubbles were removed to ensure homogeneous scaffold seeding; the vial cap was loosened and allowed to de-gas in a vacuum chamber for 10 min, a sufficient time to eliminate air bubbles without removing all dissolved oxygen in the cell slurry. Six scaffolds per well were added to a 12-well plate (Nunc). After 12 h non-adherent cells were discarded and adhered cells were cultured in iCell Cardiomyocyte media (cellular dynamics) for both iCell iPSC cardiomyocytes and primary cardiac cells. Media was changed every other day and cells were cultured in a 37 incubator with 5% supplemental CO_2_ for a maximum of 7 days.

### Gross imaging and video

A Leica M165FC dissecting microscope equipped with a DFC425 camera and Leica Application Suite software v3.8.0 was used to take bright-field and fluorescent images of the empty ECM scaffolds and Matristem^®^ (Acell, Columbia, USA) and seeded ECM scaffolds on explanted mouse heart, tissue, respectively. Videos of cell-seeded, beating ECM scaffolds were taken with an Olympus IX51microscope equipped with fluorescence and DP72 CCD camera and CellSens software v1.6.

### Mass spectrometry

#### Sample preparation

Samples of decellularized tissue solubilized in 0.1 M acetic acid were submitted to the University of Utah Health Sciences Center Mass Spectrometry and Proteomics Core (Salt Lake City, USA) for protein identification.

#### Digest of proteins in solution

Proteins from heart scaffolds were digested with TPCK-modified trypsin (Promega). Trypsin (in 50 mM ammonium bicarbonate) was added to the solution (adjusted to pH 7.9) to obtain a ratio of ~1–25 (enzyme to protein). Digest reactions were allowed to continue for overnight (at 37°C) for standard protein ID analyses.

#### Mass spectrometry

Peptides were analyzed using a nano-LC–MS/MS system comprised of a nano-LC pump (Eksigent) and a LTQ-FT mass spectrometer (ThermoElectron Corporation, San Jose, CA, USA). The LTQ-FT is a hybrid mass spectrometer with a linear ion trap used typically for MS/MS fragmentation (i.e. peptide sequence) and a Fourier transform ion-cyclotron resonance (FT-ICR) mass spectrometer used primarily for primary MS accurate mass measurement of peptide molecular ions. The LTQ-FT is equipped with a nanospray ion source (ThermoElectron Corp.). Approximately 5–20 fmoles of tryptic digest samples were dissolved in 5% acetonitrile with 0.1% formic acid and injected onto a C18 nanobore LC column for nano-LC–MS/MS and identification of peptides. The nanobore column was homemade [C18 (Atlantis, Waters Corp); 3 µm particle; column: 75 µm ID × 100 mm length] Atlantis dC18, 3 μm × 75 μm × 100 mm (Waters Corp.). A linear gradient LC profile was used to separate and elute peptides, consisting of 5–70% solvent B in 78 min with a flow rate of 350 nl/min (solvent B: 80% acetonitrile with 0.1% formic acid; solvent A: 5% acetonitrile with 0.1% formic acid). The LTQ-FT mass spectrometer was operated in the data-dependent acquisition mode controlled by Xcalibur 1.4 software, in which the “top 10” most intense peaks observed in an FT primary scan (i.e. MS survey spectrum) are determined by the computer on-the-fly and each peak is subsequently trapped for MS/MS analysis and peptide fragmentation (sequencing by collision-induced dissociation) in the LTQ linear ion trap portion of the instrument. Spectra in the FT-ICR were acquired from *m*/*z* 400 to 1,700 at 50,000 resolving power with about 3 ppm mass accuracy. The LTQ linear ion trap was operated with the following parameters: precursor activation time 30 ms and activation *Q* at 0.25; collision energy was set at 35%; dynamic exclusion width was set at low mass of 0.1 Da and high mass at 2.1 Da with one repeat count and duration of 10 s.

#### Mascot database searches

LTQ FT MS raw data files were processed to peak lists with BioworksBrowser 3.2 software (ThermoElectron Corp., San Jose, CA, USA). Processing parameters used to generate peak lists were as follows: precursor mass 351–5,500 Da; grouping was enabled allowing 5 intermediate MS/MS scans; precursor mass tolerance 5 ppm, minimum ion count in MS/MS was set to 15, and minimum group count was set to 1. Resulting DTA files from each data acquisition file were merged and the data file was searched for identified proteins against the NCBI human taxonomy sub-database, using MASCOT search engine (Matrix Science Ltd.; version 2.2.1; in-house licensed). Searches were done with tryptic specificity, allowing two missed cleavages and a mass error tolerance of 5 ppm in MS spectra (i.e. FT-ICR data) and 0.5 Da for MS/MS ions (i.e. LTQ linear ion trap). Variable modification included in the searches was oxidation of methionine, histidine and tryptophan residues. Identified peptides were generally accepted only when the MASCOT ion score value exceeded 20. Peptides identified in the MASCOT search results were all further validated by manual confirmation of molecular ions from the FTMS spectra and assigned fragment ions from the corresponding MS/MS spectra.

### Surface coatings with decellularized tissue

Glass chamber slides (Chamber slide system 154534, Nunc) were coated with decellularized cardiac tissue solubilized in 0.1 M acetic acid, diluted to 1.5 mg/ml.

Chamber slides (Chamber slide system 154534, Nunc) were coated by incubation with cardiac ECM protein solubilized in 0.1 M acetic acid (diluted to 1.5 mg/ml) overnight at 4°C and subsequently washed three times with PBS. Coated chamber slides were air-dried and sterilized by UV exposure in biosafety cabinet for 30 min.

### Mouse heart explant and in situ scaffold placement

Care of animals was in accordance with institutional guidelines. Necropsies were performed on 6 C57/BL6 mice routinely sacrificed by veterinary staff for other purposes. Hearts were aseptically removed and the pericardium was dissected away. ECM scaffolds seeded with viable cardiomyocytes and cultured for 6 days were overlaid onto the left ventricle portion of the freshly isolated hearts. Once placed, scaffolds remained untouched on this cardiac tissue surface for 3 min. After this time, forceps were used to attempt to displace the scaffold from the surface by sliding the scaffolds along heart wall.

### Confocal imaging

For viability analysis, cell-seeded ECM scaffolds were stained with Calcein AM and PI (Invitrogen, Carlsbad, USA) according to manufacturer’s instructions and imaged on their external surfaces and through their center zones. To image cell viability within scaffold centers, the scaffolds were cut transversely and placed cut-side down onto a glass slide and imaged (a second slide was held perpendicular to the first glass slide to stabilize the cut scaffold). Confocal images from 3 scaffolds and 3 images per scaffold were collected in the red and green channels. Using ImageJ (imagej.nih.gov), the number of green (Calcein AM) cells were compared to the number of red (PI) cells in each frame to obtain percent cell viability on day 7. The average and standard deviation (SD) from 9 images are displayed below.

To determine cell distribution, cells were fixed in 4% paraformaldehyde (Affymetrix, Cleveland, USA) and labeled with and 4′,6-diamidino-2-phenylindole (DAPI, Invitrogen) and rhodamine-phalloidin (Life Technologies, Carlsbad, USA) according to manufacturer’s instructions. Rabbit anti-cardiac troponin T (Abcam, Cambridge, USA 1:200), rabbit anti-connexin 43 (Abcam 1:200) and rabbit anti-*N*-cadherin (Abcam 1:200) were used with secondary antibody Donkey anti-rabbit-488 (Invitrogen, 1:500).

Cells within scaffolds were imaged on a Nikon AR1 confocal microscope. For all images, a z-series comprising 7 sections, 20 μm thick were stacked into a single image (spanning 140 μm total). A Prairie multi-photon confocal microscope was used to image the second harmonic signal of collagen from both the explanted native mouse heart and ECM scaffold placed onto the mouse heart.

### Scanning electron microscopy

Scaffolds were fixed in 4% paraformaldehyde and 2% glutaraldehyde and post-fixed in 2% osmium tetroxide, dehydrated through a series of ethanol washes, and dried with hexamethyldisilazane. The scaffolds were then sputter-coated with gold (30 s at 40 microamps) using a Pelco SC-7 autosputter coater and imaged with a SEM (FEI Quanta 600 FEG scanning electron microscope) under high vacuum.

### Optical action potential

After 6 days of culture, ECM scaffolds (3 mm in diameter, 0.3 mm thick) with a porogen size of 250 µm seeded with iCell cardiomyocytes were used for optical action potential and microelectrode array recordings. iCell media was replaced with a voltage-dependent dye solution (Infrared dye USD DI-4ANBDQBS diluted 1:1,000 in iCell media) for 7 min. After 7 min, media was replaced with dye-free iCell media. All media used was pre-warmed to 37°C. Scaffolds were transferred to an imaging chamber filled with iCell media on a Nikon microscope. Only 70 µl of media was added to keep scaffold moist, but prevent it from floating. A 50 W heat lamp was used 4 inches away from the imaging chamber to keep the cells at 37°C (a thermometer was placed in the imaging chamber to ensure a constant temperature was maintained). The heat lamp was turned off only during direct image capture.

To reduce movement artifacts, cardiomyocyte seeded scaffolds were treated with 10 µM blebbistatin (reconstituted in DMSO at 16.7 mM) diluted in HEPES to uncouple cardiomyocyte motion. Scaffolds were allowed to sit for 15 min for the uncoupler to take effect prior to imaging.

### Microelectrode array recordings

Also on day 6, another set of cell-seeded ECM scaffolds was analyzed using a custom-built microelectrode array apparatus (kindly donated by A. Moreno, University of Utah). iCell cardiomyocyte seeded scaffolds were recorded in 37°C iCell media. To confirm the electrical acuity of the cell-seeded ECM scaffolds, 1 µM isoproterenol (Hospira, Lake Forest, USA) was added and the scaffolds were subsequently recorded.

## Results

### Purified heart ECM comprises numerous proteins

**Figure 1:**
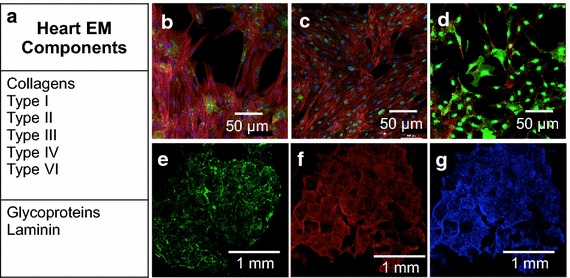
Proteins in cardiac ECM and cells. **a** Summary of primary proteins found in human heart ECM via mass spectrometry. Confocal images of human primary left ventricle cells cultured in 2D on purified human heart ECM stained for **b** connexin 43 and co-labeled with actin and DAPI, **c** troponin, and **d** *N*-cadherin and in 3D scaffolds made of human heart ECM expressing **e** connexin 43 and labeled with **f** phalloidin, and **g** DAPI.

Mass spectrometry analysis performed on purified and solubilized heart ECM proteins indicates that the highest concentration of proteins include collagen I, II, II, IV, and VI and laminin. Results are displayed in Figure 1a. The entire list of proteins detected is listed in Additional file 1: Figure S1.

### Heart matrix in 2D and 3D supports cardiac phenotype

Soluble heart ECM protein was coated onto glass slides and primary-derived cardiac cells from explanted hearts were cultured. Cells displayed non-specific staining of heart markers including connexin 43, troponin, and *N*-cadherin (Figure 1b–d). The lack of robust cardiomyocyte staining indicates a larger population of cardiac derived fibroblasts, progenitor cells, or other stromal cells and not cardiomyocytes were present. This indicated that a pure population of cardiomyocytes would need to be implemented. However, the protein did show that it supported stromal cardiac cell growth, which was promising. Human heart-derived ECM was also used to create 3D porous scaffolds and subsequently seeded with primary-derived cardiac cells. Scaffolds were co-labeled with actin, DAPI, and Connexin 43 to show cell distributions (Figure 1e–g).

### 3D porous ECM scaffolds posses large pores compared to decellularized tissue

**Figure 2:**
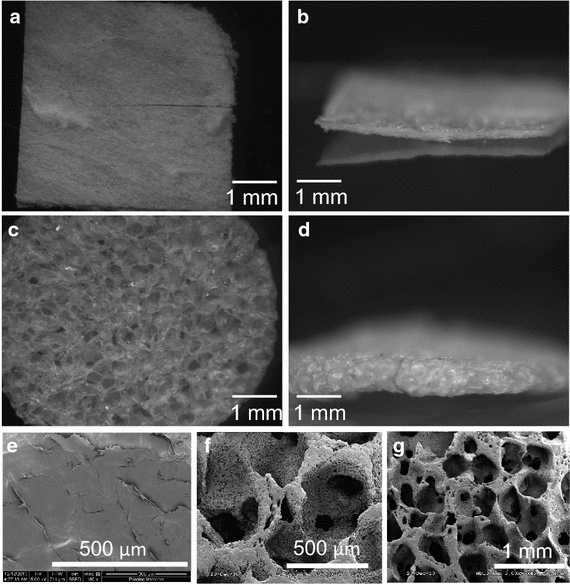
Microscopic analysis of scaffold porosity. Dissecting microscope images of **a** *top view* and **b** cross section of Matristem^®^ and a **b** *top view* and **c** and **d** cross sections of our human heart ECM scaffolds. SEM images of **e** Matristem^®^, and our PCL template scaffolds in **f** high and **g** low magnification.

Using the unique method described for processing ECM protein on sacrificial porous PCL templates, 3D matrices made of human heart protein with pore sizes of 425–500 µm were generated. These pore sizes were far larger than those seen in commercially available Matristem^®^ decellularized porcine urinary bladder matrix (UBM, Figure 2).

### Large ECM scaffold pores enable substantial cell viability

**Figure 3:**
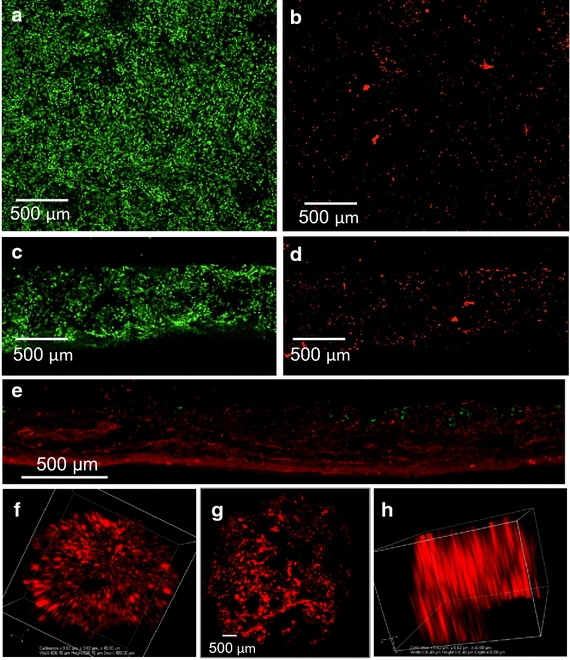
Viability of cardiac cells within scaffolds. Confocal images of *top views* of **a** viable (*green* Calcein AM) and **b** dead (*red* propidium iodide) left ventricular primary cells. Cross-sectional views of **c** live and **d** dead left ventricular primary cells. Cross-sectional view of live (*green*) and dead (*red*) left ventricular primary cells in Matristem^®^ (**e**). **f**–**h** Confocal image views of 30 stacked z-slices of human cardiomyocytes within human ECM scaffolds showing homogeneous distribution. Cardiomyocytes express red fluorescent protein under control of endogenous Myh6 promoter.

Pore sizes >425 µm allowed detection of viable seeded left ventricular primary cells throughout the 0.75 mm scaffold thickness after 7 days of culture, 62.15% ± SD 5.89% viability (Figure 3a–d). Heart ECM-derived scaffolds maintained more viable cells and improved cell distribution through a greater thickness (Figure 3c–d). Compared to commercially available Matristem^®^ scaffolds (Figure 3e) To optimize cell–cell contacts and yield a very high density of seeded cardiomyocytes, scaffolds of 0.3 mm thickness with pore sizes of 250 µm were created. A z-stack of iCell cardiomyocytes spanning 500 µm is seen within these 3D constructs (Figure 3f–h).

### iCell iPSC cardiomyocytes in 3D ECM scaffolds maintain electrical competence

**Figure 4:**
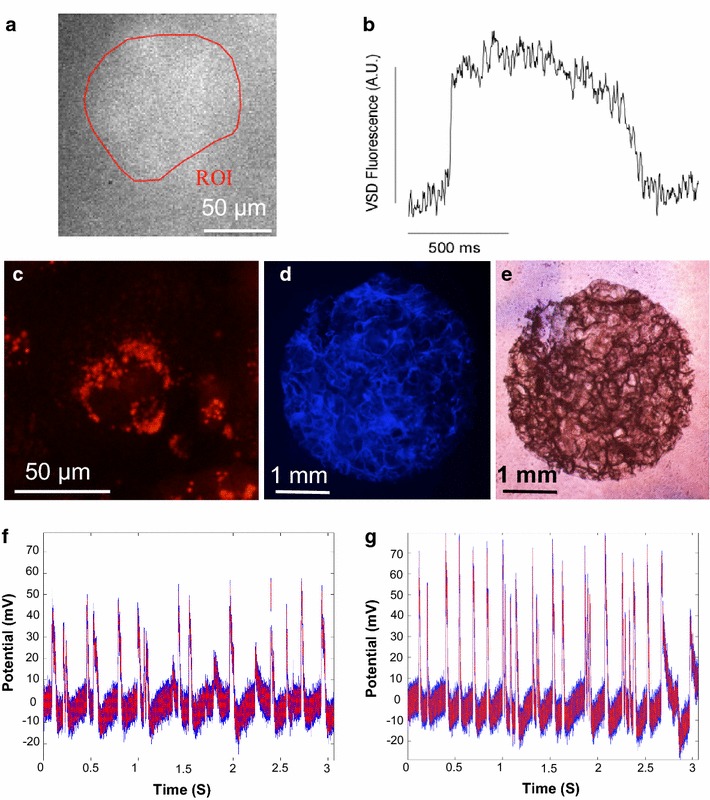
Electrical competence of cardiac cells within scaffolds. **a** Fluorescent image of voltage sensitive dye and corresponding **b** optical action potential of human cardiomyocytes within 3D human heart ECM scaffold. Still images of beating cardiomyocytes in **c** 2D culture, and in 3D scaffolds seen **d** fluorescently and **e** in Brightfield. Corresponding videos can be found in Additional files 2, 3, 4: Video 1, 2A, 2B, respectively. Cardiomyocytes maintained sufficient intercellular connections that enabled the scaffolds to contract as a single unit. Microelectrode array recordings of field potentials from human cardiomycoyte-seeded scaffolds **f** before and **g** after isoprenaline (Videos are found in Supplementary Videos 3A and 3B, respectively). Addition of isoprenaline heightened the amplitude and increased the beat rate 27%.

Seeded iCell iPSC-derived human cardiomyocytes within the ECM scaffolds were electrically competent and maintained long action potentials (Figure 4a, b) characteristic of human cardiomyocytes [43].

Day 2 post-seeding, iCell iPSC cardiomyocytes within scaffolds began to beat. By day 6, cardiomyocytes began beating synchronously both in 2D (Figure 4c; Additional file 2: Video 1) and in 3D and were able to contract the scaffold in unison (Figure 4d, e; Additional files 3, 4: Video 2A, B). This synchrony decayed after day 6 likely as cardiac fibroblasts proliferated on the ECM scaffold (data not shown). Still shots of videos taken are shown in Figure 4c–e.

iPSC cardiomyocytes within scaffolds also responded to Isoproterenol by increasing the amplitude and frequency of their beats (Figure 4f, g). Irregularities in beat rate could be due to incomplete confluency, which could allow subpopulations of cardiomyocytes to affect the consistency of the field potentials detected with the microelectrode array. Videos of this pharmacologically altered beat frequency can be found in Additional files 5, 6: Videos 3A and B.

### Cardiomyocyte adhesion behavior on ECM scaffolds

**Figure 5:**
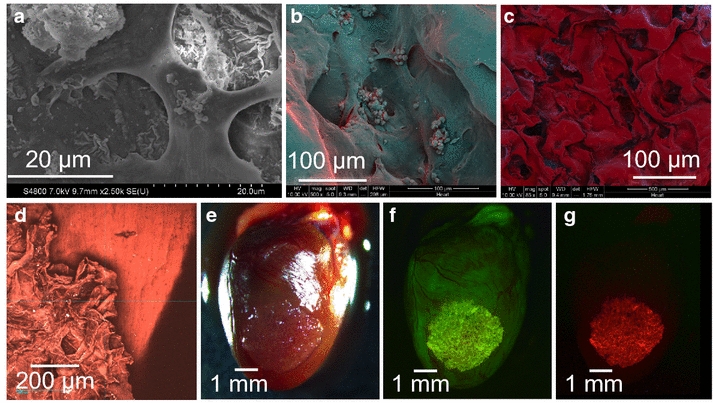
Attachment of cardiac cells on scaffolds. False colored scanning electron microscope images of human cardiomyocytes on human heart extracellular matrix scaffolds in **a** high, **b** medium, and **c** low magnifications. **d** Two photon microscope image of a scaffold overlaid over a murine heart. The second harmonic of the collagen fibrils are visualized. Dissecting microscope images (representative) of a human cardiomyocyte-seeded human ECM scaffold on an adult mouse heart: **e** shows scaffold in brightfield, **f** shows autofluorescent scaffold on a lesser autofluorescent mouse heart, and **g** shows red florescent cardiomyocytes within the scaffold. Scaffolds spontaneously fused to mouse heart within 3 min.

Cardiomyocytes readily attach to the scaffold surface as illustrated by scanning electron micrograph images (Figure 5a–c). Interestingly when the cell-seeded scaffold was placed on freshly (within 1–2 min of death) explanted murine heart surfaces, the ECM scaffolds spontaneously adhered to the murine tissue within 3 min. Once the scaffolds adhered, they could not be directionally moved or readjusted on the mouse heart tissue surface (Figure 5d–g). When cell-seeded scaffolds were placed on murine hearts preserved in saline at 4°C for 24 h, the scaffolds did not adhere and the scaffolds could easily be removed (data not shown). This suggests there is an active cell binding that occurs in the presence of live heart tissue, which could enable the scaffolds to adhere in vivo without the need for exogenous methods for adhesion. Figure 5d shows a second harmonic optical image of the scaffold adhered to the mouse heart. This optical image shows the misalignment of fiber directionality between the heart (*) and scaffold (s).

## Discussion

It is commonly believed progressive heart failure following MI is associated with adverse cardiac remodeling. Several approaches exist to promote healthy cardiac remodeling and bulk up the infarcted wall. One approach is the use of hydrogels made of synthetic polymers [44] or animal derived ECM [27, 28] that are injected into infarcted areas. This treatment modality shown to help prevent scar expansion and wall thinning, increases left ventricular ejection fraction, and improves overall cardiac function [27, 28, 44]. To provide more relevant signaling cues and avoid undesired foreign body reactions, this study focused on ECM protein, rather than synthetic polymers. Specifically cardiac ECM, pursued by several groups [27, 28, 30], was utilized to provide the most relevant biochemical signaling cues and proteins specific for heart structural and physiological function, such as collagen and laminin (Figure 1a, Additional file 1: Figure S1). Moreover, to facilitate further clinical translation and reduce potential risks associated with xenogeneic proteins such as viral disease transmission and non- homologous signaling, we utilized purified human heart ECM protein, rather than animal-sourced ECM. There are over 3,000 hearts in the US that are not used for transplant, but are used for tissue and valves. A single heart can be utilized to create scaffolds that can patch dozens of hearts. Thus, human heart ECM scaffolds are a viable option for scaffold generation clinically. Importantly, this study not only uses human ECM, but also utilizes a novel method to form human heart ECM into perhaps unparalleled highly porous, voluminous scaffolds with strikingly interconnected, uniform pores. The human heart ECM purified in this study was solubilized and coated onto glass surfaces and supported primary heart cell phenotype (Figure 1b–d), showing promise as a biomaterial.

However, ECM alone as a biomaterial has so far been shown to be insufficient in addressing degenerative cardiac tissue or restoring damaged cardiac tissue to functional form. More effective treatment strategies for cardiac repair will likely comprise tissue-specific ECM and incorporated, viable cells capable of maintaining and sustaining tissue-context-dependent phenotypes and functions. Cell therapies of diverse forms have shown limited efficacy in regenerating damaged heart tissue, in large part by providing regenerative signaling cues [4]; however, only a small portion of these delivered cells make it to or remain within the compromised tissue zone in vivo [11]. A cellular delivery vehicle that better ensures tissue site-, context-, and function-specific localization of cells, their viability, phenotype and effective integration is required. The heart-derived, porous 3D-templated ECM matrix described here shows initial promise in cardiac cellular support, exploiting xenogeneic-free purified human heart ECM with cardiac cells.

Cell sheets have been utilized to deliver functional cells to defects in the heart, but the viability of these monolayers limits sheet thickness and subsequently cell number and accompanying ECM [45]. Other groups have also pursued fibroblast-grown ECM, however these constructs are still limited to 70 μm-thick, and increasing their thickness results in poor cell viability due to poor diffusion [46]. Porous, volumetric, 3D constructs, like those pursued in this study, are an option to maximize the number of cells and ECM protein that can be delivered to a heart defect, thereby overcoming limitations encountered with current cardiac repair approaches.

Three primary methods for generating analogous 3D porous scaffolds from ECM materials are commonly employed including electrospinning, lyophilization, and decellularization. However, all three methods yield limited pore sizes, thereby decreasing the number of cells that are capable of being delivered using the scaffold. Electrospinning generally provides matrix pore sizes of 25–100 µm [47], lyophilization yields pore sizes of 10–250 µm [41] that can be highly heterogeneous [48], and decellularized constructs only have pore sizes of the absent cells (e.g., 10’s of microns). Cells entomb themselves in extracellular matrix [49], so retroactively adding back cells to such decellularized, tight matrix voids (Figure 2) is highly inefficient and nearly impossible to control well. Cell infiltration of these scaffolds can be severely limited with small matrix pore sizes. Brown et al. reported poor cell infiltration into both decellularized porcine urinary bladder matrix (UBM) and small intestinal submucosa (SIS) [38]. Commercially available decellularized UBM, Matristem^®^ compared in this study with our ECM porous scaffolds showed both poor cell infiltration (limited to ~100 µm penetration distances from the outer surfaces) and resulting viability (Figure 3). Phipps et al. seeded cells onto electrospun PCL, collagen I, and hydroxyapatite composite matrices and only were able to achieve ~100 µm of cell penetration through the thickness of their scaffolds [42]. Another group using poly polyglycolic acid (PGA) woven mesh scaffolds had far fewer cells within the center of their scaffolds even with circulating media [50, 51]. This same group began using commercially available lyophilized collagen sponges that still only contained viable cells within 100 µm thick layer around the periphery of their scaffolds [51].

The novel human heart ECM-derived porous scaffolds described in this study are capable of breaking this apparent 100 µm viability barrier. Possessing large interconnected pores, they enable complete penetration throughout 750 µm thick constructs, 7.5 times greater than Matristem^®^ (Figure 3). The scaffolds in this study also demonstrate nearly twice as many viable cells as previously reported PGA scaffolds [52]. Moreover they support homogenous cell distribution that maintains high cell viability (62.15% ± SD 5.89%) out 7 days (Figure 3), even under static cell culture conditions—without need for circulating media or complex perfusion systems. This feat is of paramount importance as an implanted construct must be able to maintain viability until host vascularization can occur. 7 days has shown to be a sufficient time period for vascularization to occur [53]. The scaffolds developed in this study support a clinically relevant number of cells for a clinically relevant period of time. Importantly, the diameter of the scaffold has no affect on viability (data not shown), enabling larger constructs that cover larger surface areas of the heart to be employed. The ECM scaffolds developed in this study utilized porous polymer templates as sacrificial scaffolds with controlled geometry and density, a process commonly used in metallurgy, enabling precise control over final ECM protein structure and porosity. These sacrificial PCL scaffolds are endowed with fine control over pore sizes using porogens. Salt porogens were used to create polymer matrix pore sizes ranging from 250 to 500 µm, far larger than those commonly seen in known commercial ECM materials (Figure 2). After porogen removal, the porous polymeric scaffolds were coated with solubilized, purified human heart ECM, using air-drying processes to bond serially deposited ECM layers together. This process of drying creates strong, pure ECM protein scaffolds without the crosslinking utilized by many current ECM scaffold technologies [39, 41]. Exogenous chemical methods crosslink ECM proteins [54] in manners distinct from native lysyl oxidase crosslinking of native proteins [55]. Chemical crosslinking alters the mechanical properties of the scaffold, reducing elasticity and compliance, and importantly reducing degradative capacity [54]. Highly degradable ECM will better expedite tissue regeneration and replacement of scaffold ECM by native ECM, a feature readily afforded by ECM scaffolds lacking chemical crosslinking. Chemical crosslinking also increases the body’s innate foreign body response, resulting in a highly oxidative cellular environment often lethal to both exogenous and endogenous cells, thus inhibiting regeneration [56]. These known deleterious effects of the host foreign body response have limited some use of scaffolds [19]. Scaffolds absent of chemical crosslinking are afforded faster degradation and reduced foreign body reaction potential, and likely improved regenerative capacity compared to commercially available and commonly used chemically cross-linked ECM scaffolds.

This study utilized heterogeneous primary cardiac cells and homogenous iPSC human cardiomyocytes seeded in novel human heart derived ECM scaffolds. Our previous work has successfully cultured stem cells on similarly constructed adipose-derived scaffolds [57], which may also be pursued for cardiac regeneration therapy. Matrix pore sizes of 425–500 µm and monolith thickness of 0.75 mm for primary heterogeneous cardiac cells provided reliable cell viability and phenotypes (Figure 3). However, this heterogeneous population of cells possessed a large amount of cardiac fibroblasts and too few functional cardiomyocytes to enable a functional beating construct. iPSC derived cardiomyocytes were then utilized as a pure enough population to potentially enable synchronous beating within the scaffolds. To optimize sufficient cell densities for cardiac synchronization, scaffolds with pore sizes of 250 microns and thicknesses of 0.3 mm were employed. These matrix fabrication conditions enabled both cell seeding and maintenance of sufficient iPSC cardiomyocyte densities to mechanically contract the entire ECM scaffold in unison (Figure 4d, e; Additional files 3, 4: 2A, B). Though contracting occurred, subpopulations of cardiomyocytes were still present in the scaffold that likely affected the complete unity of the cells within the scaffold. Lack of complete confluency and the presence of multiple pacemaker cells driving the preparation could cause irregular beating. Future work will strive to achieve total confluence and uniformity. The iPSC cardiomyocytes seeded within these same ECM scaffolds displayed electrical competence and maintained long action potentials (Figure 4a, b) consistent with human cardiomyocyte action potentials [43, 58]. Though great variability with iPSCs in culture has been seen ranging from 250 to 1,500 ms [59].

These cells also responded to beta-adrenergic agonist and cardiac chronotrope, Isoproterenol, as expected by increasing the amplitude and frequency of their beats in response to this pharmacology (Figure 4f, g; Additional file 5, 6: 3A, B). This increase in amplitude could be due to an increase in recruitment of additional beating cells or the reduction in action potential duration dispersion due to faster beating. Similar tests utilizing isoproterenol have also been utilized by other groups to prove cardiomyocyte functionality [51, 60]. These metrics indicate that both pacemaker cells and cardiomyocytes maintain many of their responsive mechanisms within these ECM scaffolds. Future studies will analyze potential arrhythmia formation post isoproterenol and how syncytium structure and anisotropic force development affect functionality.

A microscopic analysis of seeded cells on these ECM scaffolds shows cells maintain several points of adhesion to the ECM scaffold (Figure 5a–c). Interestingly, when these cardiomyocyte-seeded scaffolds were placed on freshly explanted mouse hearts, the entire scaffold spontaneously adhered to the mouse ventricle tissue within minutes, making it impossible to remove the scaffold with forceps without tearing it (Figure 5d–g). The second harmonic optical image from a two-photon microscope highlights differences in collagen fibril orientation between the scaffold and murine heart tissue (Figure 5d). Scaffolds with aligned collagen fibrils will likely beat more efficiently. Future work will be to create scaffolds with a more linear pore structure to encourage linear collagen fibril and cardiomyocyte alignment. Electrical stimulation has also be used to increase both collagen fibril alignment and ultrastructure of tissue engineered heart scaffolds, a technique employed by Vunjak-Novakovic’s group that resulted in constructs with greater ultrastructure similarity to native heart tissue [61].

## Conclusions

We have developed novel human derived scaffolds with sufficient pore size to enable the seeding and survival of a critical number of human cardiomyocytes to maintain cardiac synchrony. This construct acts as a cell delivery vehicle that can serve as a patch to treat ischemic heart tissue and directly target cells to damaged tissue sites. Furthermore these constructs are made entirely of human heart protein and human cells, and grown in a completely xeno-free system. Our process eliminates risk of xeno-disease transmission, optimizes regenerative capacity, and provides an expedited therapy for clinical translation.

## Additional files

Additional file 1:
**Figure S1.** Proteins present in human cardiac ECM. Primary protein components of decellularized human cardiac tissue identified by mass spectrometry, showing many collagens, extracellular matrix proteins, and cytoskeleon-associated proteins etc. Additional file 2:
**Video 1.** Cardiomyocytes grown in 2D on gelatin, beating synchronously. Taken with a 40X objective. Additional file 3:
**Video 2A.** Cardiomyocyte-seeded scaffolds beating the entire scaffold synchronously in A) brightfield and B) autofluorescence using a fluorescent microscope with a 10X objective. Additional file 4:
**Video 2B.** Cardiomyocyte-seeded scaffolds beating the entire scaffold synchronously in A) brightfield and B) autofluorescence using a fluorescent microscope with a 10X objective. Additional file 5:
**Video 3A.** Brightfield videos of iPS cardiomyocytes within scaffolds A) before Isoproterenol, and B) after Isoproterenol, showing increased beat rate post-treatment. A brightfield microscope with 40X objective was utilized. Additional file 6:
**Video 3B.** Brightfield videos of iPS cardiomyocytes within scaffolds A) before Isoproterenol, and B) after Isoproterenol, showing increased beat rate post-treatment. A brightfield microscope with 40X objective was utilized.

## Abbreviations

ECM: extracellular matrix
MI: myocardial infarction
3D: 3-dimensional
SDC: sodium deoxycholate
PCL: polycaprolactone
RFP: red fluorescent protein
HBSS: Hanks basic salt solution
DPBS: Dulbecco’s phosphate buffered saline
NEAA: non-essential amino acids
