# Supplementary material for: Novel xeno-free human heart matrix-derived three-dimensional scaffolds
Source: J Transl Med. 2015 Jun 18;13:194. doi: 10.1186/s12967-015-0559-0 (PMC4505384; doi:10.1186/s12967-015-0559-0)
Supplement: Additional file 1: — Figure S1. Proteins present in human cardiac ECM. Primary protein components of decellularized human cardiac tissue identified by mass spectrometry, showing many collagens, extracellular matrix proteins, and cytoskeleon-associated proteins etc. [file 12967_2015_559_MOESM1_ESM.pptx]

## Slide 1
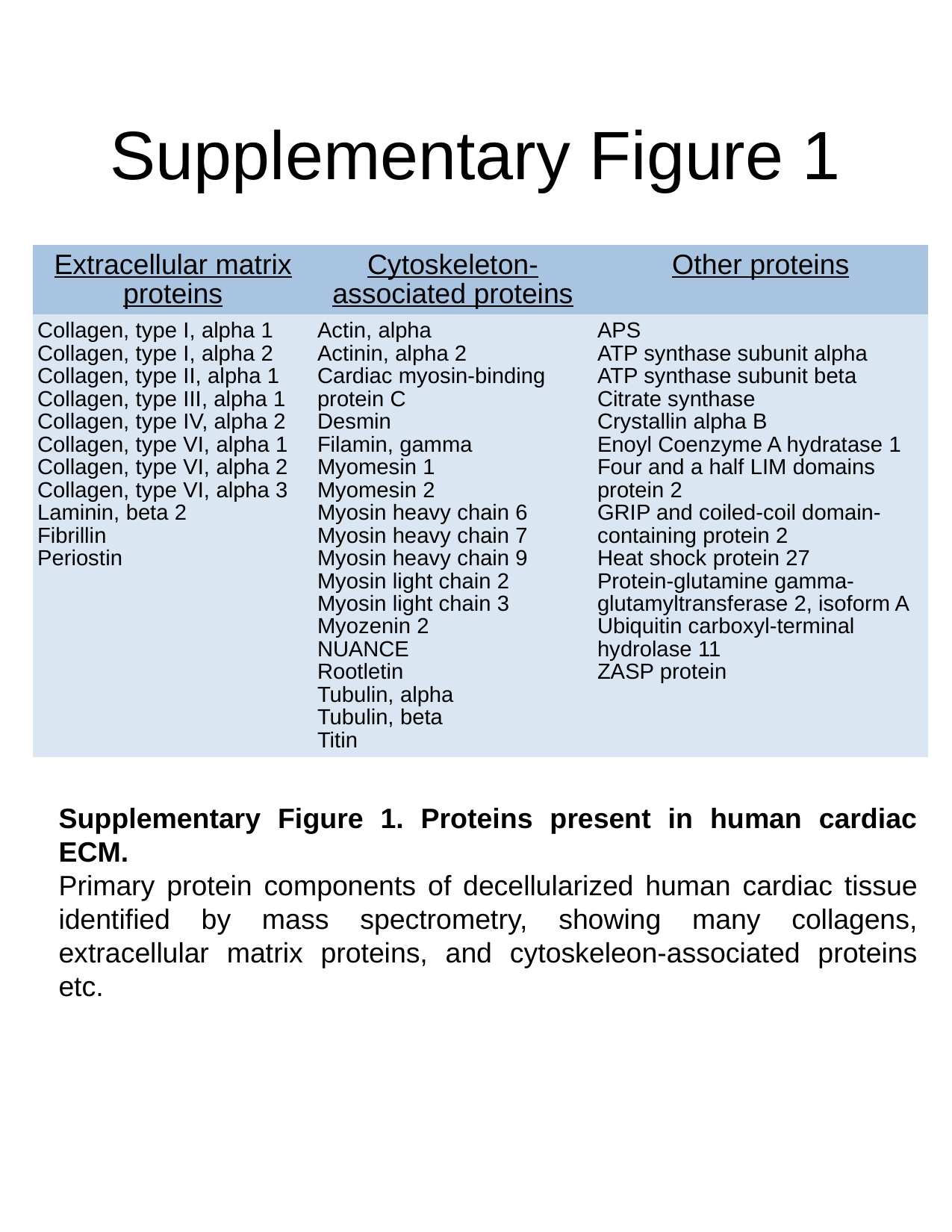

# Supplementary Figure 1
| Extracellular matrix proteins | Cytoskeleton-associated proteins | Other proteins |
| --- | --- | --- |
| Collagen, type I, alpha 1 Collagen, type I, alpha 2 Collagen, type II, alpha 1 Collagen, type III, alpha 1 Collagen, type IV, alpha 2 Collagen, type VI, alpha 1 Collagen, type VI, alpha 2 Collagen, type VI, alpha 3 Laminin, beta 2 Fibrillin Periostin | Actin, alpha Actinin, alpha 2 Cardiac myosin-binding protein C Desmin Filamin, gamma Myomesin 1 Myomesin 2 Myosin heavy chain 6 Myosin heavy chain 7 Myosin heavy chain 9 Myosin light chain 2 Myosin light chain 3 Myozenin 2 NUANCE Rootletin Tubulin, alpha Tubulin, beta Titin | APS ATP synthase subunit alpha ATP synthase subunit beta Citrate synthase Crystallin alpha B Enoyl Coenzyme A hydratase 1 Four and a half LIM domains protein 2 GRIP and coiled-coil domain-containing protein 2 Heat shock protein 27 Protein-glutamine gamma-glutamyltransferase 2, isoform A Ubiquitin carboxyl-terminal hydrolase 11 ZASP protein |
Supplementary Figure 1. Proteins present in human cardiac ECM.
Primary protein components of decellularized human cardiac tissue identified by mass spectrometry, showing many collagens, extracellular matrix proteins, and cytoskeleon-associated proteins etc.
